# Glycyrrhizic acid modified *Poria cocos* polyscaccharide carbon dots dissolving microneedles for methotrexate delivery to treat rheumatoid arthritis

**DOI:** 10.3389/fchem.2023.1181159

**Published:** 2023-05-23

**Authors:** Qi Chen, Chengyuan Wu, Siwei Wang, Qiang Wang, Peiyun Wu, Lei Wang, Peiyu Yan, Ying Xie

**Affiliations:** ^1^ Faculty of Chinese Medicine, Macau University of Science and Technology, Macao, China; ^2^ College of Pharmacy, Anhui University of Chinese Medicine, Hefei, China; ^3^ Macau University of Science and Technology, Faculty of Chinese Medicine, State Key Laboratory of Quality Research in Chinese Medicines, Macao, China; ^4^ State Key Laboratory of Dampness Syndrome of Chinese Medicine, The Second Affiliated Hospital of Guangzhou University of Chinese Medicine, Guangzhou, China

**Keywords:** Poria cocos polysaccharide, carbon dots, glycyrrhizic acid, methotrexate, rheumatoid arthritis, microneedles

## Abstract

**Introduction:** Rheumatoid arthritis is an autoimmune disease characterized by chronic joint inflammation. Methotrexate is one of the most effective drugs for rheumatoid arthritis, but the adverse reactions caused by oral methotrexate greatly limit its clinical application. Transdermal drug delivery system is an ideal alternative to oral methotrexate by absorbing drugs into the human body through the skin. However, methotrexate in the existing methotrexate microneedles is mostly used alone, and there are few reports of combined use with other anti-inflammatory drugs.

**Methods:** In this study, glycyrrhizic acid was first modified onto carbon dots, and then methotrexate was loaded to construct a nano-drug delivery system with fluorescence and dual anti-inflammatory effects. Then hyaluronic acid was combined with nano-drug delivery system to prepare biodegradable soluble microneedles for transdermal drug delivery of rheumatoid arthritis. The prepared nano-drug delivery system was characterized by transmission electron microscopy, fluorescence spectroscopy, laser nanoparticle size analyzer, ultraviolet-visible absorption spectroscopy, Fourier transform infrared spectroscopy, differential scanning calorimeter and nuclear magnetic resonance spectrometer. The results showed that glycyrrhizic acid and methotrexate were successfully loaded on carbon dots, and the drug loading of methotrexate was 49.09%. The inflammatory cell model was constructed by lipopolysaccharide-induced RAW264.7 cells. *In vitro* cell experiments were used to explore the inhibitory effect of the constructed nano-drug delivery system on the secretion of inflammatory factors by macrophages and the cell imaging ability. The drug loading, skin penetration ability, *in vitro* transdermal delivery and *in vivo* dissolution characteristics of the prepared microneedles were investigated. The rat model of rheumatoid arthritis was induced by Freund's complete adjuvant.

**Results:** The results of in vivo animal experiments showed that the soluble microneedles of the nano drug delivery system designed and prepared in this study could significantly inhibit the secretion of pro-inflammatory cytokines and had a significant therapeutic effect on arthritis.

**Discussion:** The prepared glycyrrhizic acid-carbon dots-methotrexate soluble microneedle provides a feasible solution for the treatment of Rheumatoid arthritis.

## 1 Introduction

Rheumatoid arthritis (RA) is a systemic disease characterized by inveterate inflammatory and synovium abnormal hyperplasia with unknown etiology (Aletaha and Smolen, 2018; Chen et al., 2019; Miao et al., 2021; Cush, 2022). RA generally involves the joints of the knees, hands and wrists. Severe symptoms can lead to tissue damage, joint dysfunction, infection and even death (van der Woude and van der Helm-van Mil, 2018; Lin et al., 2020). At present, the therapy of RA usually includes subcutaneous injection or per os antirheumatoid drugs, non-steroid anti-inflammatory drugs and glucocorticoids (Fraenkel et al., 2021; Wu et al., 2021). Methotrexate (MTX) is the first choice for the treatment of RA (Abbasi et al., 2019). Tablets are the main dosage forms of MTX in clinical use (Emery et al., 2020; Schiff and Sadowski, 2017). Long-term oral administration of MTX may lead to gastrointestinal adverse reactions (Artacho et al., 2021), myelosuppression (Tu et al., 2020), and abnormal liver and kidney function (Alfaro-Lara et al., 2019). Poor compliance also hinders the research progress of RA cure (Blair and Deeks, 2017). Transdermal administration has many potential dominance and is an ideal supersede route of administration. Containing avoiding the first-pass effect, reducing the frequency of administration and reducing the side reaction on the gastrointestinal tract (Amjadi et al., 2018). Traditional drug percutaneous absorption rate is very restricted due to skin barrier. Microneedles (MNs) can penetrate the stratum corneum and are an array of many micron-sized tiny needles (Caffarel-Salvador and Dinnely, 2016; Wang et al., 2017; Zhao et al., 2022). As a new type of drug delivery system, MNs mainly create multiple instantaneous microchannels by reversibly piercing the stratum corneum barrier to deliver drugs without damaging blood vessels and stimulating nerves, which can also improve patient acceptability. (An and Liu, 2017). At present, most of the studies on MTX MNs are used alone to treat RA. There are few reports on the combination with other anti-inflammatory drugs, and the function is single.

Carbon dots (CDs) are an accidental discovery of carbonaceous nanomaterials with a size of less than 10 nm (Ansari et al., 2021; Dugam et al., 2021). CDs preparation method is simple, the surface is rich in carboxyl, hydroxyl and other functional groups, low cytotoxicity, is described as a “carbon core” with surface functional groups (Janus et al., 2019). As an excellent and stable photoluminescence nanoparticle, CDs have the advantages of high biocompatibility (Iravani and Varma, 2020), wide excitation spectrum (Feng et al., 2018), sensitive optical environment (Zhang et al., 2022), low toxicity and wide combination with other nanoparticles compared with previous quantum dots and organic fluorescent dyes. It can not only specifically deliver drugs to targeted sites, but also observe cell status and drug delivery process through fluorescence imaging (Zhao et al., 2019), so they are often selected as ideal carriers for drug delivery processes and biomedical applications (Li et al., 2020; Wang et al., 2022). Glycyrrhizic acid (GA) is one of the most important active ingredients in licorice. It is a triterpenoid saponin with anti-inflammatory, anti-viral, anti-cancer and hepatoprotective effects (Sun et al., 2019; Richard, 2021). GA can effectively inhibit the growth and replication of various viruses by stabilizing cell membrane and immune regulation, and has a specific effect on the expression and activity of specific virus-related enzymes (Zhao et al., 2021). GA can also be used as a multi-functional drug carrier to enhance the activity of other drugs (Selyutina and Polyakov, 2019). Chunxian Piao (Piao et al., 2022) prepared curcumin-loaded acidic nanoparticles by self-assembly method. The results showed that compared with glycyrrhizic acid + curcumin, curcumin-loaded glycyrrhizic acid nanoparticles had higher anti-inflammatory effects *in vitro-in vivo* by reducing pro-inflammatory cytokines. In the animal model Glycyrrhizic acid-Curcumin r abated inflammatory repercussion decidedly compared with GA, Curcumin, and Glycyrrhizic acid + Curcumin. Compared with the use of a single drug, the combination of two or more anti-rheumatoid drugs can improve the therapeutic efficiency. The combination of GA and MTX provides a new method for the treatment of RA.

In this study, CDs were synthesized by one-step hydrothermal method, modified by GA and loaded with anti-RA drug MTX to prepare Glycyrrhizic Acid-Carbon Dots-Methotrexate (GA-CDs@MTX) nano drug delivery system with fluorescence and double anti-inflammatory effects, high biocompatibility and low toxicity; Hyaluronic acid (HA) was combined with nano drug delivery system to prepare biodegradable Glycyrrhizic Acid-Carbon Dots-Methotrexate Microneedles (GA-CDs@MTX MNs) for the treatment of RA by percutaneous administration. After implantation into skin tissue, GA-CDs@MTX can be controlled to be released from MNs with the degradation of hyaluronic acid MNs patch. Firstly, we used various microscopic and spectral techniques to characterize the prepared GA-CDs@MTX, and explored the therapeutic effect of GA-CDs@MTX on lipopolysaccharide-stimulated RAW264.7 cell inflammation model. Then the drug loading, skin penetration, transdermal delivery *in vitro* and dissolution *in vivo* of MNs were studied systematically. The rat model of RA with complete Freund‘s adjuvant was used to monitor the restraining effect of GA-CDs@MTX MNs on foot swelling and the expression of pro-inflammatory cytokines to evaluate the *in vivo* efficacy of GA-CDs@MTX MNs.

## 2 Materials and methods

### 2.1 Materials

*Poria cocos* polysaccharide (Lot: CHB190302) was got from Chengdu Chroma-Biotechnology Co., Ltd. Methotrexate (Lot: 2014286), (1-(3-dimethy-laminopropyl)-3-ethylcarbodiimide hydrochloride) (EDC) (Lot: K1710148), N-hydroxyl succinimide (NHS) (Lot: J1718011) and ethylenediamine were purchased from Aladdin Chemistry Co. Glycyrrhizic acid (Lot: A25GS146530) and 0.4% trypan blue solution was gained from Shanghai Yuanye Biotechnology Co., Ltd. Complete Freund's Adjuvant (Lot: 102821220531) and Penicillin-Streptomycin Solution (100×) was purchased from Shanghai Beyotime Biotechnology Co., Ltd. Hyaluronic acid (HA, Mw = 100000–200000, Lot: C14200311) was acquire ed from Shanghai Macklin Biochemical Technology Co., Ltd. Lipopolysaccharide (LPS, *Escherichia coli* 0555:B5) was purchased from biosharp Co., Ltd. CCK8 was purchased from Shanghai dojindo Co., Ltd. DMEM medium was purchased from Nanjing wisent corporation Co., Ltd. Fetal bovine serum purchased from HyClone, United States ELISA kits were obtained from Shanghai Mlbio Co., Ltd.

The MNs were made by a 10 × 10 array Poly-dimethylsiloxane (PDMS) MNs mold (purchased from China Taizhou Microchip Pharmaceutical Technology Co., Ltd.) with a tip height of 750 μm and a base width of 340 μm. HA-based soluble MNs was prepared by two-step centrifugation Dong et al., 2018; Roh et al., 2021). A HA aqueous solution (250 mg mL^−1^) was Dumped in the PDMS molds, followed by centrifugal treatment (4000 r min^−1^) for 10 min. Then, the mold was placed in a dry dish overnight at room temperature. The MNs were carefully removed from the mold, sealed and stored in a dry dish at room temperature and away from light. In order to encapsulate MTX and GA-CDS@MTX nanoparticles into MNs patches, MTX and nanocomposites were added to HA aqueous solution with a final concentration of MTX of 50 mg mL^−1^, respectively. The MNs were observed using an electron microscope and photographed at 45° and 90°, respectively. All the tips of the MNs were removed and dissolved in water. The drug content determined was the drug loading of the MNs.

Mouse macrophage RAW264.7 cell line was obtained from Cell Resource Center, Shanghai Institutes for Biological Sciences, Chinese Academy of Sciences. SD rats purchased from Liaoning Changsheng Biotechnology Co., Ltd. All animal experiments were allowed to the Experimental Animal Management Committee of Anhui University of Traditional Chinese Medicine and carried out in secundum with the guidelines for the care and use of experimental animals.

### 2.2 Apparatus and characterization

The morphology of the GA-CDs@MTX was observed by transmission electron microscope (TEM) operated. Surface charges (zeta potentials) tested at 25°C by zetasizer nano zs90 (Malvern Instruments, United States). The structural types of CDs were characterized by X-ray diffraction (XRD). The elemental composition and elemental coordination of CDs were analyzed by X-ray photoelectron spectroscopy (XPS) (Shimadzu Corporation of Japan). UV-vis spectra were measured with a Analytik Jena SPECORD S600 UV spectrophotometer. Fourier transform infrared spectra (FT-IR) were recorded using Nicolet 6700 (Thermo Scientific, United States). The NMR hydrogen spectroscopy measurements was observed by Varian UNITY INOVA400 (400 MHz, United States) and ACE200 (200 MHz, Germany). Thermal behavior before and after MTX loading with DSC 200 F3 Maia (NETZSCH, Germany). Fluorescence spectra were recorded with a Fluorescence spectrophotometer F-4600 (Hitachi, Japan).

### 2.3 Synthesis of CDs

The preparation method of CDs adopts the method previously reported by the author’s research group (Huang et al., 2022). The CDs were synthesized through one-step hydrothermal method. 0.45 g of *Poria cocos* polysaccharide was solubilized in 30 mL of ultrapure water, and 5.0 mL ethylenediamine solution was added. After ultrasonic stirring, the mixture was taken into a high-pressure reactor and reacted at 200°C for 5 h. Cooling to room temperature, when the solution turns dark brown, indicating that CDs were successfully prepared. The solution was efferenced at 10,000 rpm for 10 min to remove the deposition. Finally, the supernatant was dialyzed in ultrapure water with a dialysis bag (molecular weight cut off: 1,000 Da) for 24 h (the dialysate was replaced every 6 h) to remove impurities, freeze-dried, and stored in 4°C refrigerator away from light. Finally, the CDs solution was freeze-dried in vacuum to obtain solid powder.

### 2.4 Synthesis of GA-CDs

GA-CDs were synthesized by amide reaction between active -NH_2_ on the surface of CDs and -COOH on GA. 20 mg GA was resolved in 8 mL PBS (pH 7.4), stirred and dissolved completely. 4 mL aqueous solution containing 26 mg EDC and 15.6 mg N-hydroxyl succinimide (NHS) was successively added to the GA solution, stirred violently at room temperature and reacted 12 h. Then 2 mL CDs solution was puted to the reaction solution and reacted for 24 h at indoor temperature and dark environment. In order to remove excess unreacted GA, dialysis bags was used for 24 h. The purified GA-CDs was freeze-dried to solid and stored in 4°C refrigerator away from light.

### 2.5 Synthesis of GA-CDs@MTX nano drug delivery system

The 5 mg GA-CDs powder was dissolved in 10 mL ultra-pure water and gently shaken to fully dissolve. A certain amount of MTX powder was diluted with PBS buffer (pH 7.4), and gently shaken to make it fully dissolved. Under the condition of stirring, the MTX solution was puted to the GA-CDs solution, and the GACDs@MTX complex solution was obtained by stirring overnight, and then the GA-CDs@MTX complex solution was placed in a dialysis bag (Mw = 1000Da). Dialysis in ultrapure water for 24 h to remove free MTX, freeze-dried solution to obtain GA-CDs@MTX composite powder, 4°C cold storage in dark.

### 2.6 MTX-loading and release

Determination of actual drug loading of GA-CDs@MTX by High-Performance Liquid Chromatography (HPLC, Thermo Scientific Dionex UltiMate 3000). According to “*The Chinese Pharmacopoeia 2020 edition,*” acetonitrile-7.0% citric acid solution-2.0% anhydrous disodium hydrogen phosphate solution (10 : 10: 80) was selected as the fluid-phase. Chromatographic column: COSMOSIL 5C18-MS-II; detection wavelength: 302 nm; column temperature: 30°C; flow rate: 1 mL·min^-1^; injection volume: 10 μL. 10 mg GA-CDs@MTX complex powder was accurately weighed, placed in a 50 mL measuring bottle, added 20 mL methanol, and ultrasounded for 30 min. Mobile phase constant volume, shake sampling. After centrifugation at 12,000 rpm for 10 min, the clear supernatant was used as the test solution and injected for determination according to the above chromatographic conditions. Then, the load capacity (LC) were accounted using the following formulas:

$$LC\;\left(\%\right)=\frac{Mass\;of\;drug\;encapsulated}{Mass\;of\;nanoparticles}\times 100$$

The *in vitro* release of the drug was investigated under pH 5.0 and pH 7.4 conditions. In short, GA-CDs@MTX solution was dialyzed for 36 h at 37°C. The concentration of the solution was detected at a predetermined time and the drug release efficiency was calculated. The efficiency of drug release was calculated by

$$Drug\;release\;\left(\%\right)=\frac{weight\;of\;drug\;released}{total\;weight\;of\;original\;drug-free\;drug}\times 100$$

### 2.7 *In vitro* cell experiment

#### 2.7.1 Cell culture

RAW264.7 cells were cultured in Dulbecco’s Modified Eagle’s Medium (DMEM) high glucose medium including heat-inactivated fetal bovine serum (FBS) and penicillin and streptomycin. The incubator culture conditions were set to 5% CO_2_/air, 37°C, and the medium was renewal every other day. The growth status of cells was observed daily.

#### 2.7.2 Nitric oxide assay

RAW264.7 cells were inoculated into 96-well plates at a density of 1 × 10^5^ cells/well. Different concentrations of lipopolysaccharide (LPS, 0, 100, 250, 500, 1,000 ng·mL^-1^) were incubated with RAW264.7 for 12 and 24 h at 37°C in a CO_2_ incubator, respectively. Cell culture medium was collected, and the concentration of nitric oxide secreted by cells was detected by NO kit. Briefly, after centrifugation of the medium, 50 μL of the supernatant was reacted with 100 μL of Griess reagent at room temperature for 10 min, and then the absorbance of the solution was measured at 540 nm using a microplate reader (Molecular Devices SpectraMax i3x).

#### 2.7.3 Cytotoxicity assay

The cytotoxicity of CDs, GA-CDs, GA-CD@MTX and free MTX against RAW264.7 cells were estimated by the CCK8 assay. The cells were inoculated into 96-well plates, cultured overnight, and incubated with LPS for 24 h. Different concentrations of nanoparticles were incubated with cells for a period of time. After the cells were treated with 10% CCK8 reagent, the wavelength of the microplate reader was adjusted to 450 nm, and the absorbance was measured.

#### 2.7.4 *In vitro* cell imaging studies

The fluorescence imaging of cells treated with GA-CDs and GA-CD@MTX was made on by an inverted fluorescence microscope (Leica DMi8). RAW264.7 cells were seeded into 12-well plates at a density of 1 × 10^5^ cells/well. Then, cells were incubated with LPS (1,000 ng·mL^-1^) for 24 h at 37°C in a CO_2_ incubator. A certain concentration of CDs GA-CDs and GA-CD@MTX was added in each set of cells followed by incubation for 6 h, respectively. After that, the cells were washed by PBS to remove extracellular GA-CDs/GA-CD@MTX. It was immobilized with 4% paraformaldehyde at room temperature for 10 min, and finally sealed with anti-fluorescence quencher for fluorescence inverted microscope observation. In addition, the uptake of GA-CDs@MTX by RAW264.7 cells stimulated by lipopolysaccharide within 6 h was investigated by the same method mentioned above.

#### 2.7.5 *In vitro* anti-inflammatory bioactivit of GA-CD@MTX

RAW264.7 cells were inoculated in 96-well plates. LPS and GA-CD@MTX were co-incubated with RAW264.7 cells for 24 h, and then the cell supernatant was collected. The levels of inflammatory factors (TNF-α, IL-6, IL-1β) were determined by ELISA.

### 2.8 *In Vivo* skin insertion tests

#### 2.8.1 *In Vivo* skin insertion capability

The ability of MNs to penetrate the skin was studied by trypan blue staining. In short, the MNs were first pressed on the skin by hand, removed after 5 min, and then stained with 4% trypan blue reagent for 2 min. After removing the excess dye and washing with water for 3 times, the stained skin was visualized by bright field microscopy.

#### 2.8.2 Acute skin irritation test of MNs

After MNs administration, the recovery of skin was observed to evaluate the skin irritation of MNs. Before the experiment, rats were paralysed by abdominal cavity injection of pentobarbital sodium (50 mg·kg^-1^). The MNs were treated for 5 min, and the electron microscope was used to photograph the administration site before administration and 0, 5, 10, 20, 30, 45, 60 min after administration.

#### 2.8.3 *In Vivo* dissolution of MNs

The dissolution of MNs *in vivo* was studied. The hair on the back of rats was removed before administration. MNs were removed at different times (0, 2, 5, 10 min) after administration, and then MNs were observed from the side by electron microscope.

### 2.9 *In Vitro* drug release of MNs

The transdermal *in vitro* release of GA-CDs@MTX was analyzed by Franz diffusion cell (Logan Instruments). Remove the hair from the back skin of the rat. After the rats were anesthetized, the back skin was removed, the subcutaneous tissue and fat were removed, and the saline was washed. The GA-CDs@MTX soluble MNs was pressed into the skin and fixed on the receiving chamber. The temperature was maintained at 37°C ± 1 °C and the receiving medium (PBS, pH 7.4) was steadily agitated at 600 rpm. The PBS solution (1 mL) was taken at a predetermined time point and 1 mL of new PBS was added to keep the volume unchanged. The concentration of MTX in the samples was detected by HPLC, and the cumulative release percentage of MTX from MNs was calculated.

### 2.10 Therapeutic effects of the MNs

The pharmacodynamics of GA-CDs@MTX MNs was studied. Rheumatoid model was induced by injecting 0.15 mL complete Freund‘s adjuvant (CFA) into the right hind foot of rats. On the 18th day after modeling, the rats were randomly divided into normal group, model group, oral MTX group, oral GA-CDs@MTX group, MTX MNs group and GA-CDs@MTX MNs group. The rats were administered once every 3 days, and the toe volume of the rats was recorded. After 21 days of administration, the rats were sacrificed and blood was taken. The levels of TNF-α, IL-6 and IL-1β in serum were measured by ELISA kit.

### 2.11 Statistical analysis

All other experiments were repeated at least 3 times except for the other experiments. One-way ANOVA was used for statistical comparison between groups. Significant differences between or among groups were indicated by *p < 0.05, **p < 0.01, ***p < 0.001 and ****p < 0.0001, ns: nonsignificant, respectively.

## 3 Results and discussion

### 3.1 Characterization

**FIGURE 1:**
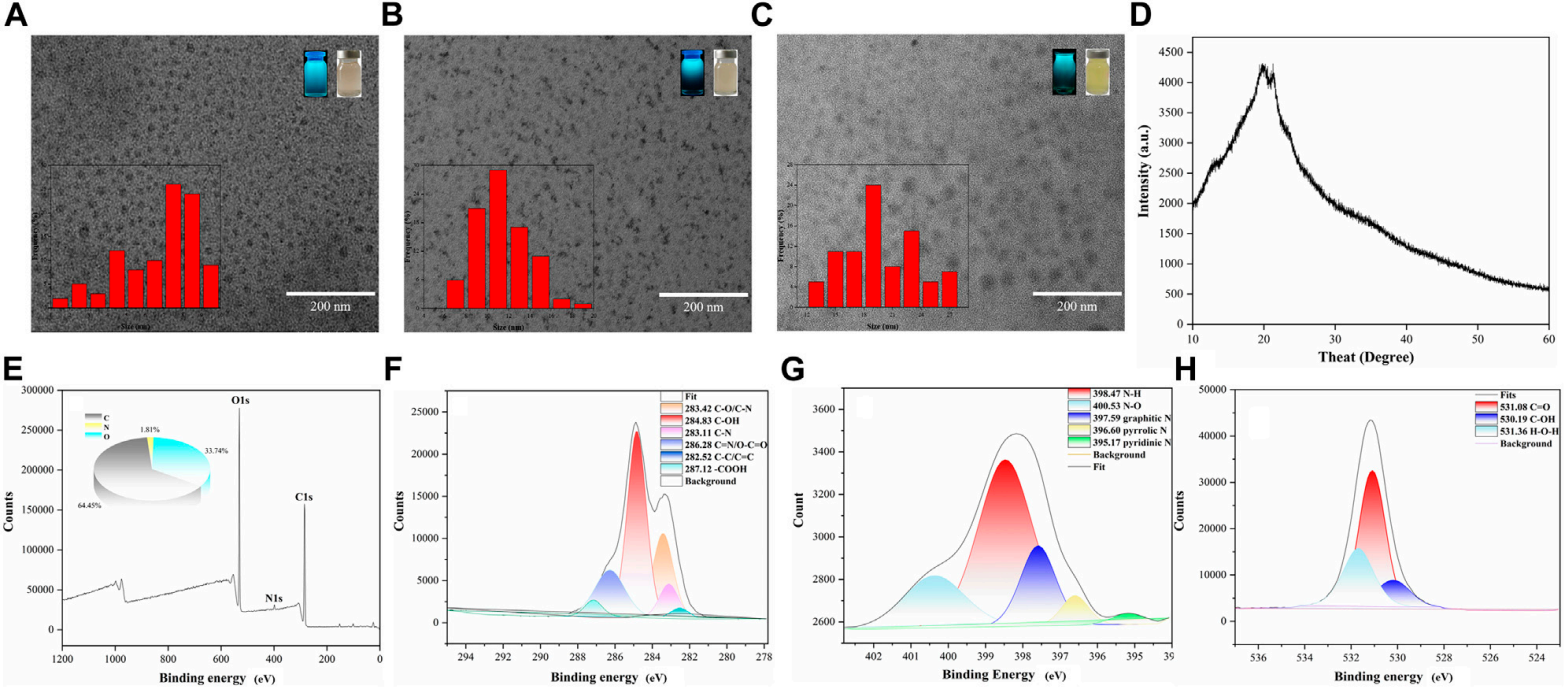
Transmission electron microscopy images of CDs **(A)**, GA-CDs **(B)** and GA-CDs@MTX **(C)**, The illustrations are particle size distribution, morphology of solution under fluorescent lamp and ultraviolet lamp; **(D)** XRD spectrum of CDs; X-ray photoelectron spectroscopy survey spectrum of CDs **(E)**, and XPS spectrum of C_1s_ region **(F)**, N_1s_ region **(G)**, and O_1s_ region **(H)**.

The morphology, size and electric potential of CDs, GA-CDs and GA-CDs@MTX were characterized by TEM and Malvern laser particle size analyzer. As shown in Figure 1A, the CDs is a spherical monodispersed particle with a uniform size and an average particle diameter of 7.20 nm. The particle size of GA-CDs was 11.40 nm. The average particle size of GA-CDs@MTX drug loading system is about 19.77 nm (Figure 1C), which is slightly larger than that of GA-CDs. The larger particle size may be caused by the connection of MTX to the carbon surface. CDs, GA-CDs, GA-CDs@MTX emit blue fluorescence under irradiation with a 365 nm UV lamp (Figures 1A–C). The XRD pattern (Figure 1D ) shows that the diffraction peaks of CDs are located at 22.76° and 22.89°, indicating that it is not a complete crystal and belongs to an amorphous structure. XPS was used to further verify the surface element characteristics of CDs. In the XPS total spectrum (Figure 1E), the three peaks at 284.8,398.22 and 531.16 eV correspond to C_1s_, N_1s_ and O_1s_, respectively, clearly indicating that CDs contain C, N and O, and the atomic ratio is C:N:O = 64.45:1.81:33.74. Figures 1F–H are the peak fitting diagrams of C_1s_, N_1s_ and O_1s_ of CDs, respectively. It can be seen from the figure that CDs have hydroxyl (−OH), carboxyl (−COOH), amide group (−NH_2_) and other groups.

**FIGURE 2:**
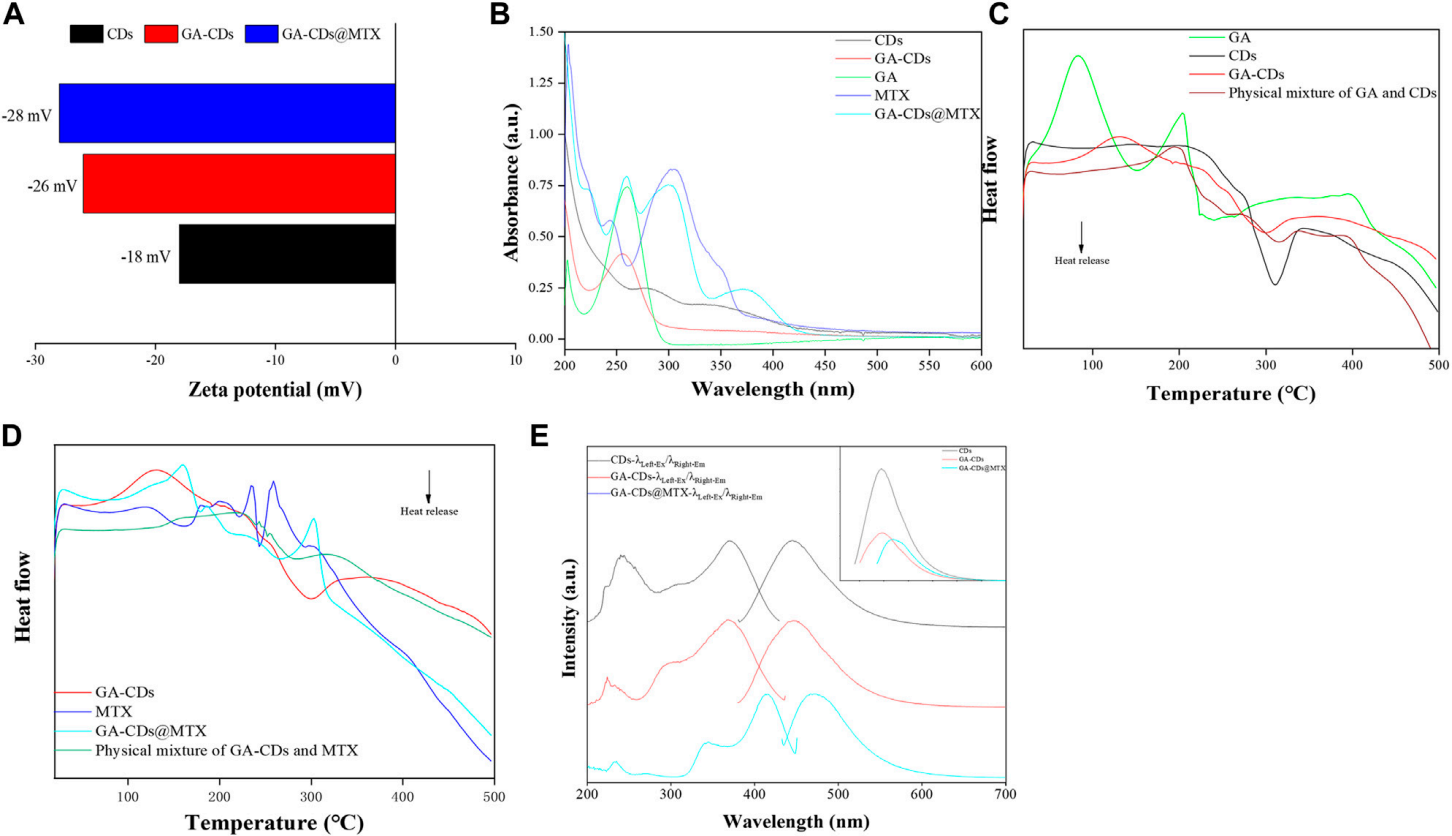
Characterization of CDs, GA-CDs and GA-CDs@MTX: **(A)** Zeta potential; **(B)** UV-vis absorption spectrum; **(C)** Differential scanning calorimetry (DSC) thermograms of GA-CDs; **(D)** Differential scanning calorimetry (DSC) thermograms of GA-CDs@MTX; **(E)** Fluorescence spectrum, The upper right illustration shows the fluorescence intensities of CDs, GA-CDs, and GA-CDs@MTX.

The surface charges (zeta potentials) of GA-CDs and GA-CDs@MTX was detected that is −26 mV and −28 mV, respectively, which is an important variables for the long-term stability (Figure 2A). As shown in Figure 2B, the states of CDs, GA, GA-CDs, MTX, GA-CDs@MTX in solution can be detected from the UV-Vis spectra. CDs has two characteristic absorption peaks at 287 nm and 344 nm, and GA has strong UV absorption at 202 nm and 260 nm. From the UV spectra of GA-CDs nanocomposites, GA is well supported on CDs. The characteristic peak of GA in 260 nm shifts left (red shift) to 255 nm, which may be caused by the interaction between ground state electron donor and acceptor (Zhang et al., 2010). Forasmuch as the presence of heteroaromatic pterine chromophore, MTX solution strongly absorbs ultraviolet light at 244 nm, 305 nm and 350 nm wavelengths. The absorption spectrum of GA-CDs@MTX solution shows three shoulder peaks at the above wavelengths, which is shifted (blue shift and red shift) compared with free MTX, which may be attributed to the strong π-π^*^ stacking interaction between MTX and GA-CDs. For example, after loading on GA-CDs, the peaks of MTX at 244 nm, 305nm and 350 nm moved to 260, 301 and 376 nm. These results show that GA-CDs and MTX molecules form a complex in solution (Arsalani et al., 2019).

The DSC curve of GA-CDs, physical mixture of GA and CDs, GA, CDs are shown in Figure 2C. CDs has exothermic peak at 311 °C and GA has two endothermic peaks at 82°C and 204°C. Compared with GA and CDs, the original peak of GA-CDs disappears and two endothermic peaks appear at 130°C and 330°C. The DSC spectra of GA-CDs@MTX complex, MTX and GA-CDs physical mixing, GA-CDs and MTX are shown in Figure 2D. A broad endothermic peak (121 °C) and two narrow endothermic peaks (235°C and 259°C) appeared in the peak of MTX. The peak shapes and characteristic peaks of MTX and GA-CDs in the DSC spectrum of the physical mixture of MTX and GA-CDs were reflected, and it could be observed that MTX and GA-CDs changed their structures almost independently with the change of temperature. However, the characteristic endothermic peak of MTX disappeared in the GA-CDs@MTX complex. It shows that the distinguishing existence of GA-CDs@MTX complex is different from that of MTX, GA-CDs and their physical mixture in thermal analysis, which on the other hand reflects the formation process of the complex between MTX and GA-CDs. Some weak intermolecular forces may occur, and the single or comprehensive interaction between them promotes the formation of the complex, such as hydrogen bond, van der Waals force, electrostatic force and so on. The optical properties of CDs, GA-CDs and GA-CDs@MTX were characterized by fluorescence spectrophotometer (Figure 2E). The results showed that with the modification of GA and loading of MTX, the fluorescence intensity of the nano-drug-loaded system decreased gradually, which was consistent with the fluorescence decline of CDs, GA-CDs and GA-CDs@MTX solutions in Figures 1A–C under 365 nm UV light. It is worth noting that when GA-CDs and MTX form a complex, the emission wavelength is red-shifted (wavelength increases), which may be due to the increase of conjugated structure when GA-CDs is loaded with MTX.

**FIGURE 3:**
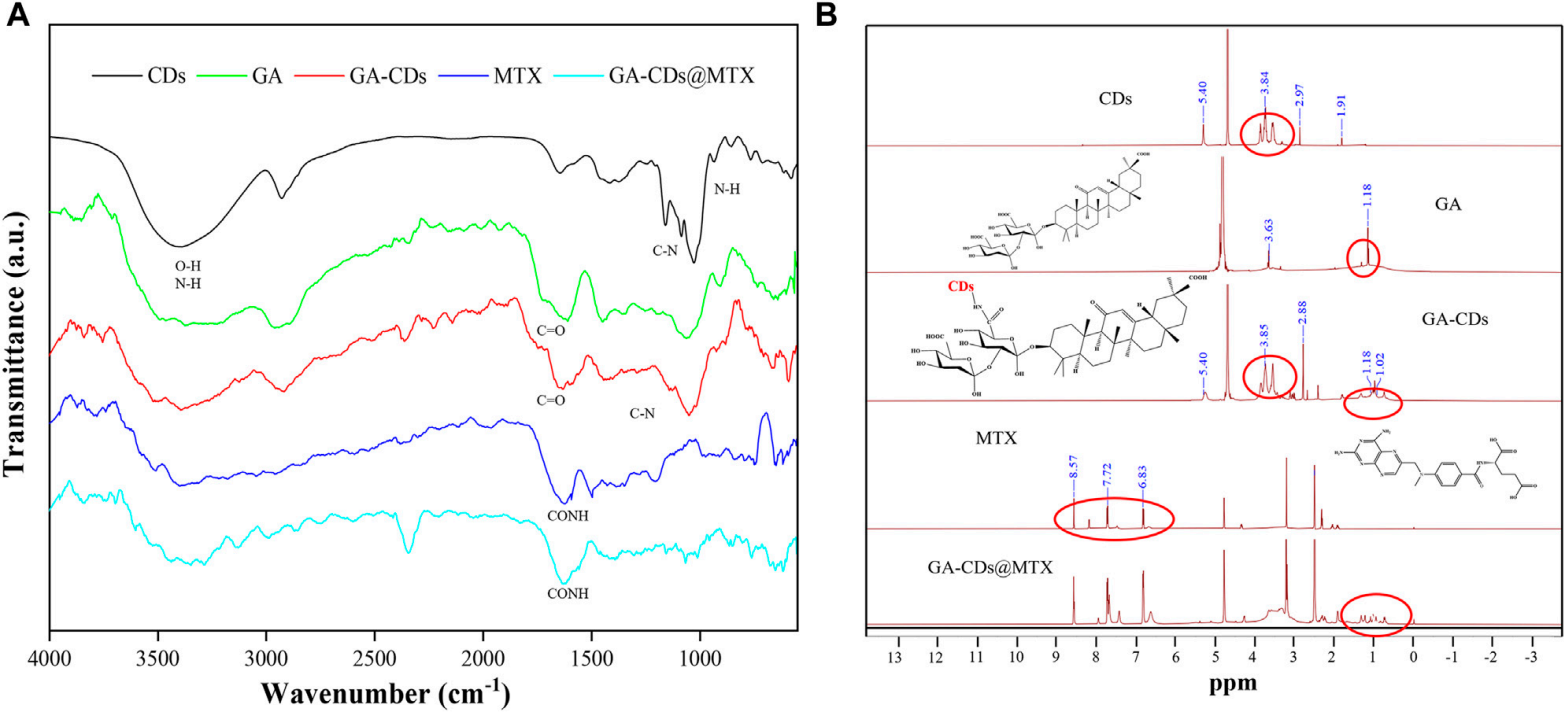
**(A)** FT-IR spectra of GA-CDs and GA-CDs@MTX. **(B)** ^1^H NMR of GA-CDs and GA-CDs@MTX.

The FT-IR spectra of CDs, GA, GA-CDs, MTX, GA-CDs@MTX showed many common characteristic peaks (Figure 3A). The characteristic peaks of the CDs are the stretching vibrations of the O-H and N-H of 3387 cm^-1^, the stretching vibrations of the C-H bonds of 2,926 cm^−1^, and the COO- stretching vibrations at 1,641 and 1,412 cm^−1^. The C=N and N-H stretching vibrations at 1,581 cm^−1^ and 851 cm^−1^, and the C-O stretching vibrations at 1,024 cm^−1^. When GA was modified on CDs, the characteristic peak of 851 cm^−1^ (N-H) shifted, and the stretching vibration of the amide absorption peaks at 1,653 cm^-1^ (C=O) and 1,291 cm^−1^ (C-N) increased, which indicated that GA was successfully modified on CDs. GA-CDs@MTX not only has the same absorption peak as GA-CDs, but also shows the characteristic band of MTX at 1,620 cm^−1^ (=CONH). The N-H stretching vibration of amino group is enhanced at 3343 and 3436 cm^−1^, and the characteristic peak of MTX is also observed at 649 cm^−1^. These observations confirm that CDs successfully loaded MTX (Khodadadei et al., 2017; Hsin et al., 2020). ^1^H NMR was used to further prove the chemical structure (Figure 3B). The characteristic signal peaks at *δ* = 5.40, 3.84 and 2.97 ppm in the CDs spectrum were present in the GA-CDs spectrum. In addition, there is a characteristic hydrogen peak of cycloalkanes (*δ* = 1.18 ppm) is the same as that of GA in the GA-CDs spectrum. This shows that GA has been connected to CDs by amide bond. In the ^1^H NMR spectrum of MTX, 6.83 and 7.72 ppm are the characteristic chemical shifts of benzoyl group in MTX, and *δ* = 8.57 ppm is the characteristic chemical shift of hydrogen atom in 2,4-diamino-6-pteridine group. The above characteristic peaks of MTX also exist in the spectrum of GA-CDs@MTX. In addition, the characteristic peaks of GA-CDs *δ* = 3.85 and 1.18 ppm also appeared in the spectra of GA-CDs@MTX. The experimental results based on FT-IR and ^1^H NMR spectra confirmed that the GA-CDs@MTX was successful synthesized.

### 3.2 GA grafting amount

 The absorbance of GA-CDs and CDs at 260 nm was measured. The results are shown in Supplementary Figure S1 and Supplementary Figure S2. The grafting amount of GA in GA-CDs was calculated to be 50.48%.

### 3.3 MTX loading and release studies

The predominantly features of the drug delivery system were explored for GA-CDs@MTX to confirme their loading and releasing efficiency. PBS with pH 5.0 and 7.4 was selected for dialysis solutions according to the presence of RA microenvironment and physiological environment in normal tissues. Supplementary Figure S3 is the HPLC chromatogram of MTX. The cumulative release rate of the drug was calculated according to the data measured by HPLC, and the results are shown in Supplementary Figure S4. The release efficiency at pH 5.0 was higher than that at pH 7.4. The release curve of both increased rapidly within 0–4 h, and then the rate of increase gradually slowed down. The final cumulative release was measured at 36 h. The cumulative release of GA-CDs@MTX was 92.0% at pH 5.0, and 65.5% at pH 7.4. The above results indicate that the prepared GA-CDs@MTX nanosystem effectively prolongs MTX release and improves the environmental selectivity of the drug.

### 3.4 Cell experiment

#### 3.4.1 Inflammation cell model

**FIGURE 4:**
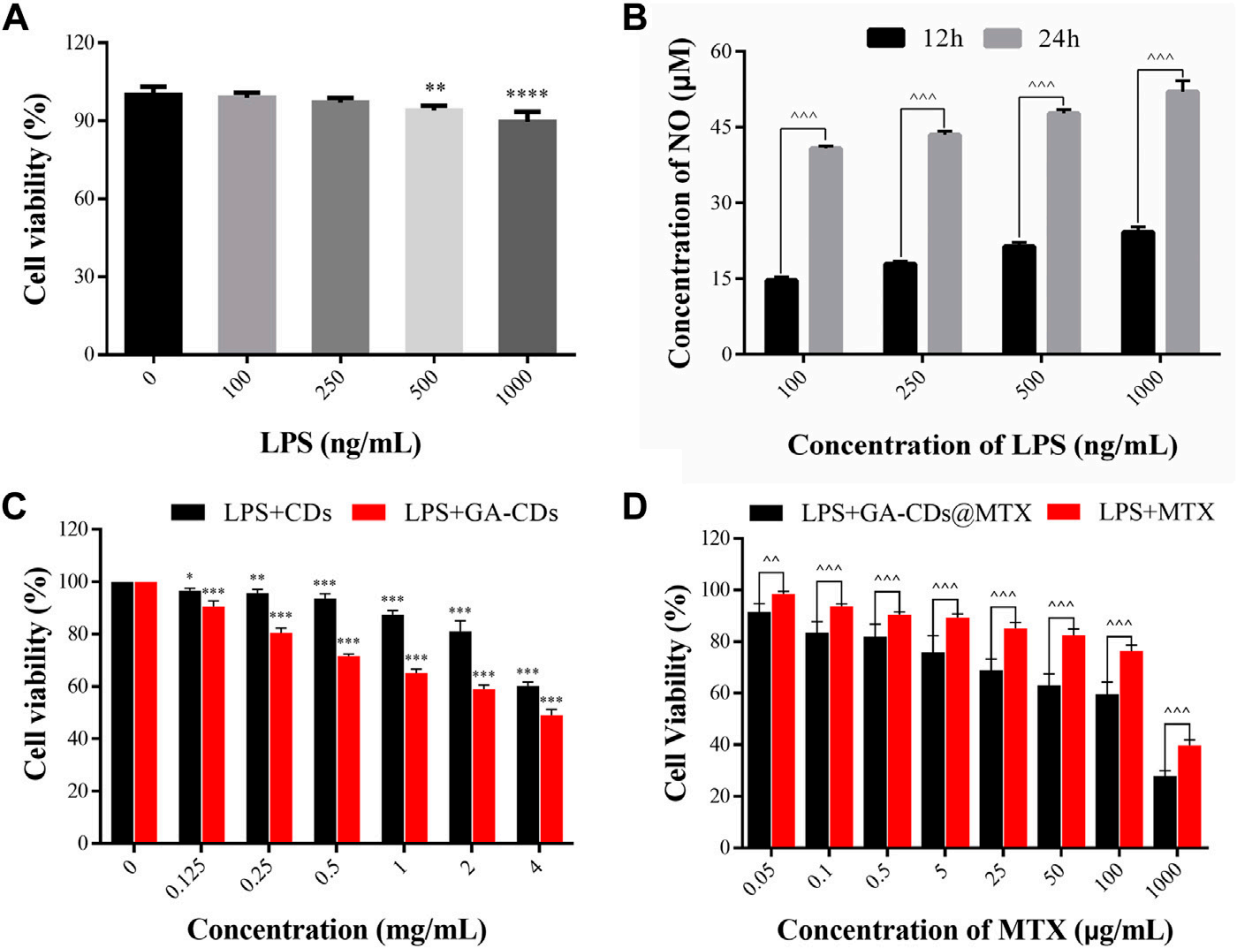
**(A)** Cell viability of LPS-treated RAW264.7 macrophages. **(B)** Concentration of NO in RAW264.7 stimulated with different concentrations of LPS at different time (12 and 24 h). **(C)** *In-vitro* cytotoxicity of CDs and GA-CDs after 24 h incubation time in LPS-treated RAW264.7 cells. **(D)** Cytotoxicity of free MTX and GA-CDs@MTX (mean ± SD, *n* = 6). *p < 0.05, **p < 0.01, ***p < 0.001, ****p < 0.0001 versus 100%; ***^^*** *p* < 0.01, ***^^^*** *p* < 0.001 between groups, ns: nonsignificant, respectively.

The construction of inflammatory cell model is mainly to use different levels of LPS to stimulate RAW264.7 cells, through the NO kit to investigate the level of NO secreted by RAW264.7 cells under the action of different LPS content, and therefore select the appropriate conditions for the construction of cell model. The cell viability of different concentrations of LPS is shown in Figure 4A. RAW264.7 cells were treated with different concentrations of LPS, and the concentration of NO in the supernatant was detected at 12 h and 24 h, respectively, the results are shown in Figure 4B. It can be seen from Figure 4B that the amount of NO secreted by RAW264.7 increased significantly with the increase of endotoxin treatment time, and showed a concentration dependence (*^^^p* < 0.001). When the concentration of lipopolysaccharide was 1000  ng·mL^-1^ and the action time was 24 h, the amount of NO secreted by cells was significantly higher than that of other low concentrations of LPS. Therefore, we choose the concentration of LPS as the action time of 1,000 ng·mL^-1^ for 24 h as the basic method to construct the inflammatory cell model.

#### 3.4.2 Cytotoxicity

It can be seen from Figure 4C that when the concentration was less than 2 mg·mL^-1^, the inhibitory effect of CDs on LPS-induced RAW264.7 cells was small after incubation for 24 h, and the cell activity was greater than 80%. When the concentration of GA-CDs was lower than 0.25 mg·mL^-1^, the cytotoxicity was lower. It is worth mentioning that at the same concentration, the cell activity of GA-CDs group was lower than that of CDs group (**p < 0.01), which may be caused by the anti-inflammatory effect of GA. RAW264.7 cells were incubated with a series of GA-CDs@MTX and MTX solutions with the same MTX concentration for 24 h after LPS induction, and the corresponding cell viability was detected by CCK8 method. The results are shown in Figure 4D. The overall trend of the inhibitory effect of GA-CDs@MTX and MTX on LPS-induced RAW264.7 cells was similar. Both of them had obvious MTX concentration-dependent cytotoxicity. With the increase of MTX concentration, the cell survival rate gradually decreased. In addition, the cell viability of GA-CDs@MTX group was significantly lower than that of MTX group (*^p* < 0.05).

#### 3.4.3 *In Vitro* fluorescence bioimaging

**FIGURE 5:**
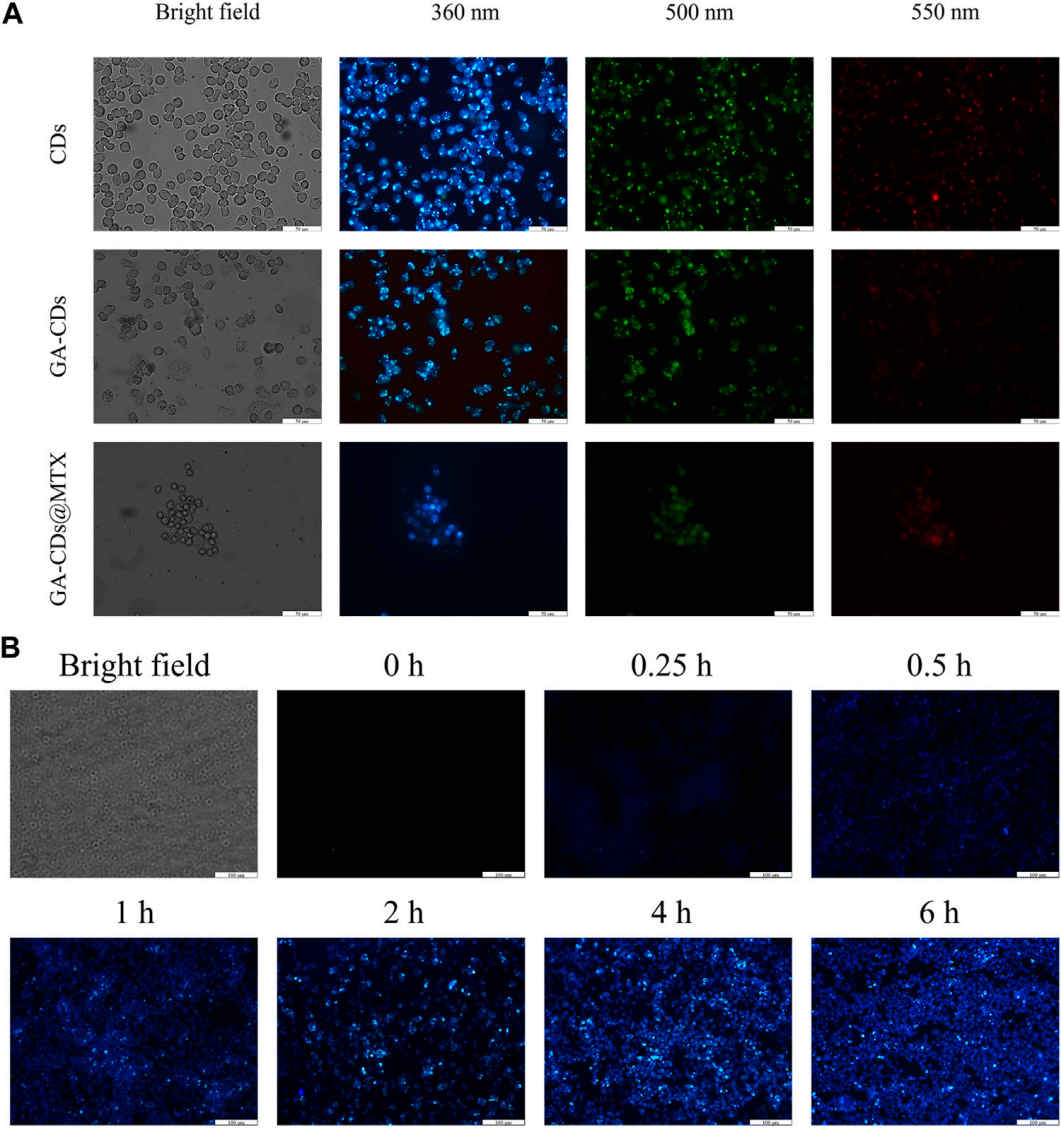
**(A)** Fluorescence microscopy images of LPS-treated RAW264.7 cells obtained under a bright field; excited at 340 nm, 495 nm and 550 nm after incubation with CDs, GA-CDs and GA-CDs@MTX for 12 h. The scale bar stands for 50 μm. **(B)** The different time points fluorescence images of LPS-treated RAW264.7 cells after incubation with GA-CDs@MTX and free doxorubicin, respectively. Scale bar = 100 μm.

The imaging behavior of the prepared GA-CDs@MTX in cell imaging was studied. As shown in Figure 5A, CDs, GA-CDs and GA-CDs@MTX were co-cultured with LPS-induced RAW264.7 cells for 6 h, and cell fluorescence images were taken under a fluorescence inverted microscope. Under laser irradiation, CDs, GA-CDs and GA-CDs@MTX-treated cells all showed strong blue fluorescence. The imaging results of GA-CDs@MTX after incubation with LPS-induced RAW264.7 cells for 0.25, 0.5, 1, 2, 4, 6 h are shown in Figure 5B. From the diagram, it can be seen that at 0.25 h, LPS-induced RAW264.7 cells showed a weak blue fluorescence signal, and the blue fluorescence signal was significantly enhanced with the increase of incubation time.

#### 3.4.4 Detection of inflammatory factors

**FIGURE 6:**
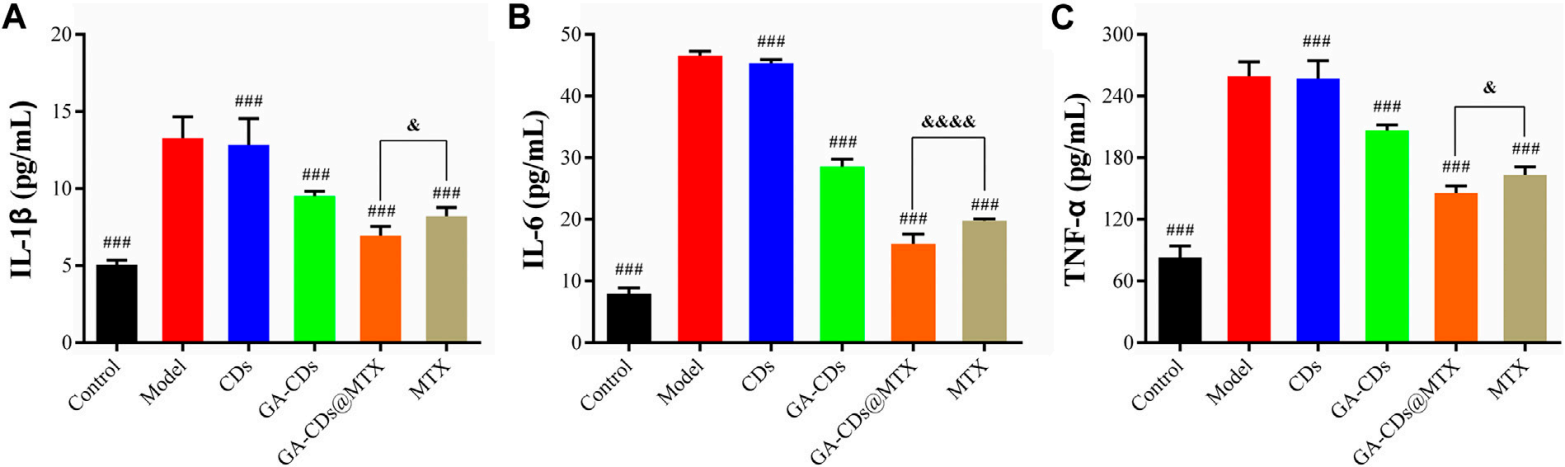
Expression of inflammatory mediators in LPS-treated RAW264.7 cells with or without CDs, GA-CDs, GA-CDs@MTX of **(A)** IL-1β, **(B)** IL-6 and **(C)** TNF-α. ^*###*^ *p* < 0.001 versus model, *&p* < 0.05, ^*&&&&*^ *p* < 0.0001 between groups, respectively.

IL-1β, TNF-α and IL-6 play an important role in joint destruction and synovial hyperplasia of RA and promote inflammatory response. We used LPS to induce RAW264.7 cells to construct an inflammatory cell model and secrete pro-inflammatory cytokines. Compared with the control group, the levels of pro-inflammatory factors (IL-1β, IL-6 and TNF-α) in the model group were significantly increased (*###p* < 0.001). After being treated with GA-CDs@MTX nanocomposites for 24 h, the cytokine level was remarkably reduced (*&p* < 0.05) (Figures 6A–C).

### 3.5 MNs fabrication and characterization

**FIGURE 7:**
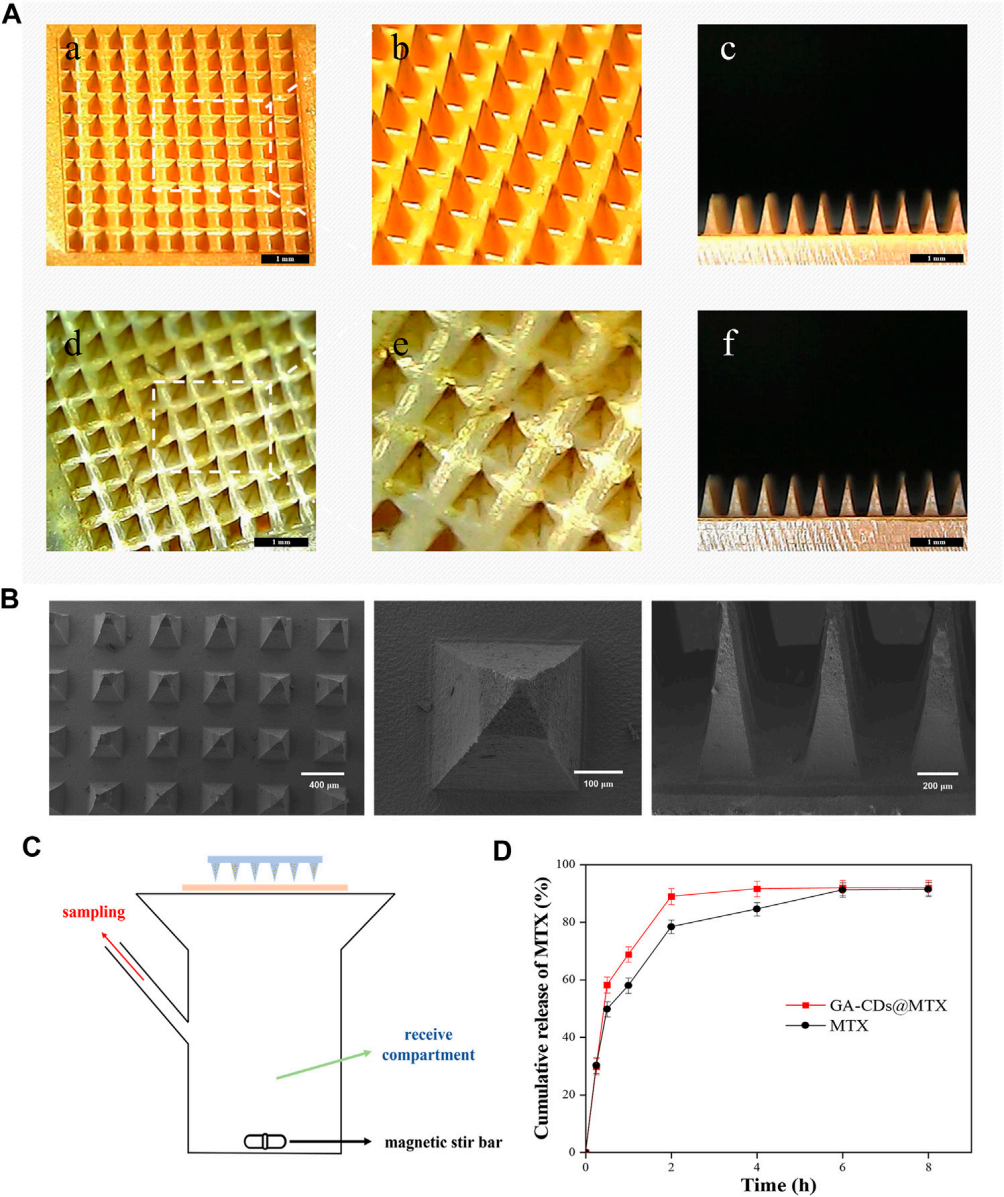
**(A)** Characterization of the MTX-loaded MNs patches. Representative dermoscopy images of the (a, b, and (c) MTX, (d, e, and (f) GA-CDs@MTX MNs patches taken at 45° and 90°. **(B)** Scanning electron microscopy images of GA-CDs@MTX MNs. **(C)** Schematic illustration of Franz diffusion cell in the study of transdermal MTX delivery. **(D)** *In vitro* permeation profile of MTX through the rats skin from the MNs patch with GA-CDs@MTX and free MTX. Data are presented as mean ± SD (*n* = 4).

GA-CDs@MTX MNs and MTX MNs was prepared using a two-step needle preparation method. The morphology of the MNs was characterized using a microscope. Because of the large cross-sectional area and small aspect ratio, the mechanical strength of the MNs with four pyramid shape is stronger than that of the Mns with cone shape (Davis et al., 2004). For this reason, we chose a MNs mold with a pyramid shape as the MNs mold for this experiment. The images of final MNs patches are shown in Figure 7A, each MNs patch consisted of 100 (10 × 10) needles with ∼340 μm width, ∼750 μm height, and ∼600 μm of center space between adjacent needles. The shape of the MNs is quadrangular cone, the tip is small and sharp, and the size distribution is uniform. The drug loading of MTX in MNs was determined by HPLC: the drug loading of MTX MNs was 71.56 μg and that of GA-CDs@MTX MNs was 70.05 μg. Figure 7B is Scanning electron microscopy images of GA-CDs @ MTX microneedles.

The rat skin was fixed on the Franz diffusion cell (Figure 7C) to investigate the efficiency of transdermal release of MTX by MNs. Figure 7D shows the cumulative release rate of MTX. It can be seen from the figure that the cumulative release rate of the drug was as high as 58% within 30 min. After that, the release rate gradually slowed down, reaching 68% and 88% at 1 and 2 h. The cumulative release amount remained almost unchanged after 4 h, and the final cumulative release rate was about 92%. The cumulative release of MTX was less than the drug content in the microneedle, which may be caused by the drug residue in the skin.

### 3.6 Skin insertion tests

#### 3.6.1 Insertion properties of and acute skin irritation test of MNs

**FIGURE 8:**
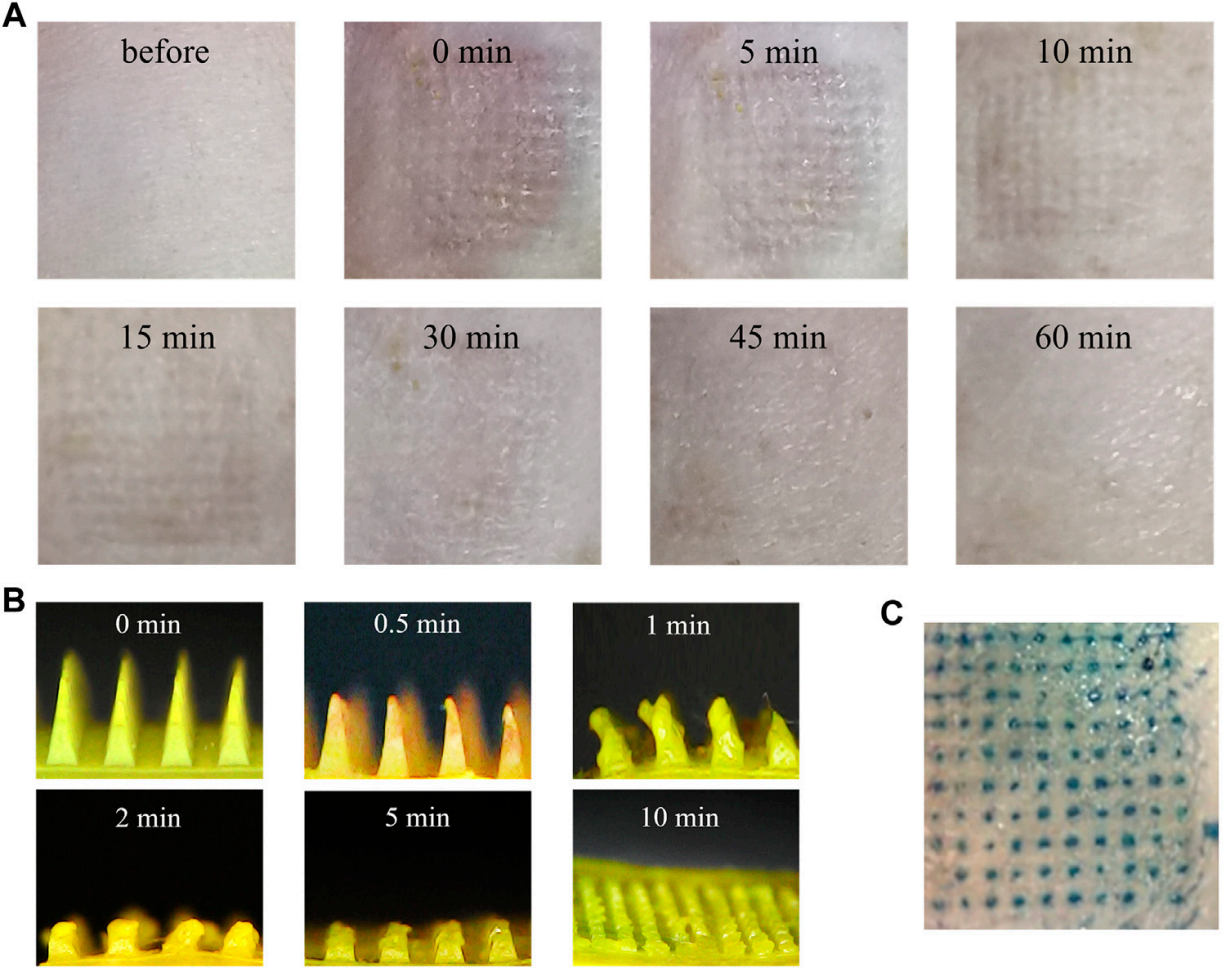
*In vivo* skin insertion evaluation of the MNs. **(A)** Dermoscopy images of MNs treated rats dorsal skin at different time intervals. **(B)** Optical microscopy images of GA-CDs@MTX MNs from a side view before and after insertion for 0, 0.5, 1, 2, 5, and 10 min into the back of the rats *in vivo*. **(C)** Optical image of rats skin after MNs were applied.

Whether the prepared MNs can penetrate the skin was studied. After MNs administration, there were obvious microporous arrays on the skin. The recovery process was photographed by an electron microscope. As shown in Figure 8A, the pinholes on the skin gradually disappeared and were almost invisible after 1 h. In addition, there was no obvious irritation such as erythema and swelling on the skin surface. GA-CDs@MTX MNs were implanted into rat skin. After trypan blue staining, obvious pinholes were observed (Figure 8C).

#### 3.6.2 *In Vivo* dissolution of MNs

The MNs on the rat skin were removed at a predetermined time to observe the dissolution of the needle tip. Figure 8B is the side view of the undissolved part of MNs. It can be seen from the figure that in the initial stage, the sharp tip of MNs becomes blunt after insertion into the skin, and the tip gradually dissolves. MNs were completely dissolved within 10 min, which may be due to the water solubility of hyaluronic acid.

### 3.7 Therapeutic effects of MNs

**FIGURE 9:**
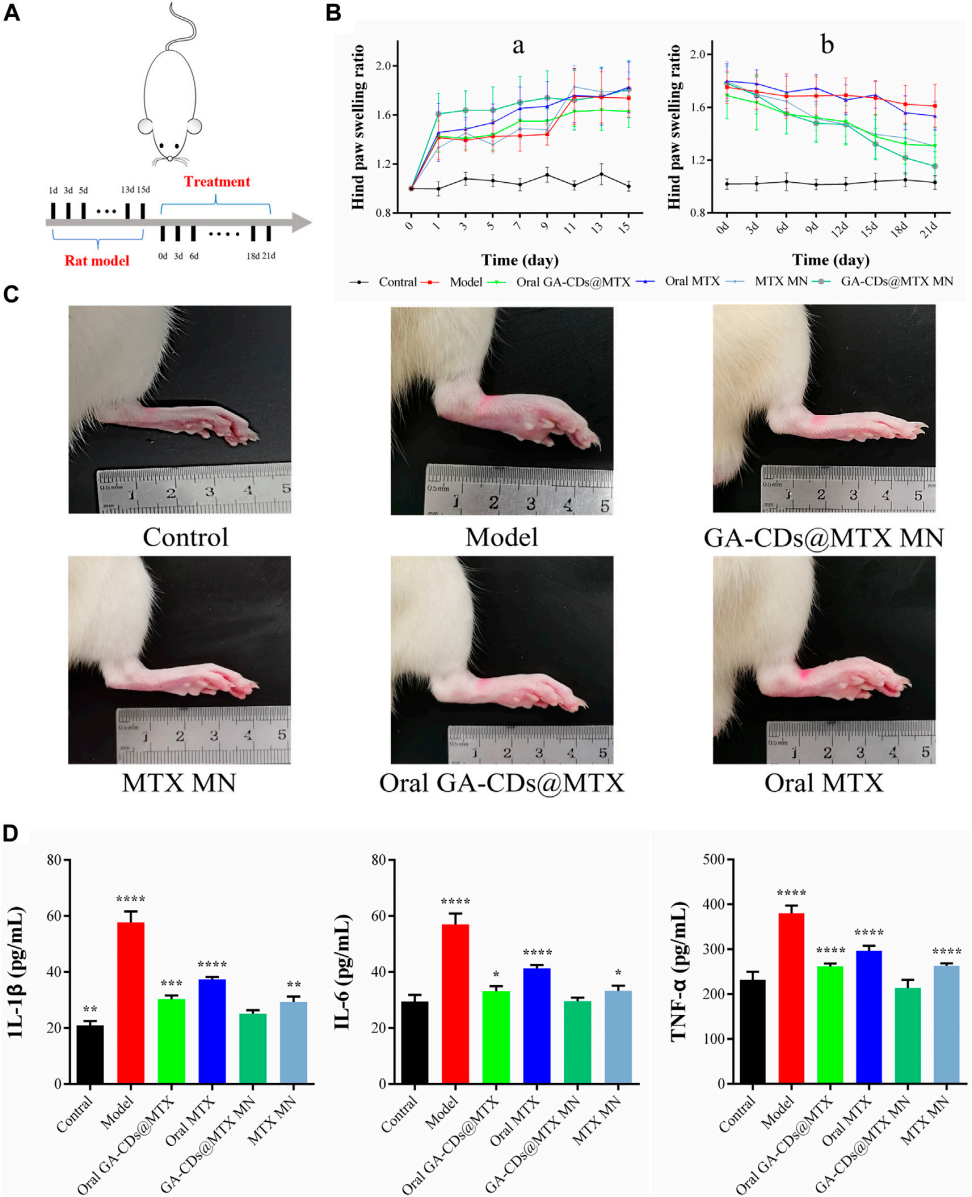
**(A)** Timeline of rats experiment. **(B)** Paw swelling ratio and of rats receiving different treatments in 21 days. **(C)** Hind paw profile images of each group after 7 days. **(D)** TNF-α, IL-6 and IL-1β concentration of rats after different treatments. ***^^^*** *p* < 0.001 versus Contral; *###p* < 0.001 versus Model; * *p* < 0.05, ** *p* < 0.01, *** *p* < 0.001, ****p <0.0001 versus GA-CDs@MTX MNs, respectively.

Oral MTX is one of the most common ways to treat RA. We compared the efficacy of oral MTX, oral GA-CDs@MTX, MTX MNs and GA-CDs@MTX MNs. Treatment was performed three times a week according to the timeline shown in Figure 9A. Figure 9B is the toe volume change of rats in the process of modeling (a) and administration (b). As shown in the figure, after subcutaneous injection of Freund‘s complete adjuvant toe, except for the blank group, the toe scores of RA rats in the other groups increased sharply. After administration, untreated RA rats maintained higher toe volume. The toe volume of rats in the oral MTX group changed little, and the degree of foot swelling in the oral GA-CDs@MTX group was lower than that in the control group. MTX MNs group showed better and faster reduction effect on foot swelling, which may be due to the avoidance of first-pass effect and sustained release effect. It is worth noting that the oral GA-CDs@MTX group showed similar efficacy as the MTX MNs group. As expected, the GA-CDs@MTX MNs treatment displayed the best therapeutic effect, and the paw swelling symptom almost disappeared after 21 days (Figure 9C). Cytokines play an important regulatory role in the process of RA synovitis and joint destruction. In this experiment, the levels of pro-inflammatory cytokines (TNF-α, IL-1β, IL-6) in the blood were detected by ELISA kit to evaluate the *in vivo* efficacy of different administration methods. The results showed that the levels of IL-1β, TNF-α and IL-6 were significantly decreased after MNs administration of GA-CDs@MTX compared with other groups (Figure 9D). This indicates that GA-CDs@MTX MNs have a better therapeutic effect on RA, which is the result of the transdermal administration of MNs, the passive targeting of rheumatoid microenvironment by GA-CDs, and the synergistic anti-inflammatory effect of GA and MTX.

## 4 Conclusion

In summary, we have successfully synthesized a nano-drug delivery system with dual anti-inflammatory effects and self-fluorescence. Based on this, soluble MNs were prepared for the treatment of RA. GA was modified onto the CDs by amide reaction, and then MTX was successfully loaded by π-π stacking, and the drug loading rate was 39.2%. Finally, a soluble MNs was prepared with hyaluronic acid as the base material. *In vitro* cell experiments showed that when the concentration of CDs was less than 2 mg·mL^-1^, the cell survival rate remained above 80%. Compared with free MTX, GA-CDs@MTX had a stronger inhibitory effect on LPS-induced RAW264.7 inflammatory cells, and reduced the levels of pro-inflammatory factors TNF-α, IL-6 and IL-1β more significantly (*p < 0.05). Skin puncture experiments showed that the prepared GA-CDs@MTX MNs could penetrate the skin, release drugs and achieve transdermal administration. Taking adjuvant-induced arthritis rats as the model, GA-CDs@MTX MNs had stronger efficacy than oral MTX and MTX MNs, reduced the inflammatory response of RA, significantly reduced foot swelling of RA rats, and achieved good therapeutic effect. This work provided a valuable MNs-assisted transdermal delivery approach, opens a new method of RA treatment.

## Data Availability

The original contributions presented in the study are included in the article/supplementary material, further inquiries can be directed to the corresponding authors.
