# Supplementary material for: Glycyrrhizic acid modified Poria cocos polyscaccharide carbon dots dissolving microneedles for methotrexate delivery to treat rheumatoid arthritis
Source: Front Chem. 2023 May 23;11:1181159. doi: 10.3389/fchem.2023.1181159 (PMC10243470; doi:10.3389/fchem.2023.1181159)
Supplement: Supplementary file 1 [file DataSheet1.docx]

**Supplementary Material**

**Glycyrrhizic acid modified Poria cocos polyscaccharide carbon dots dissolving microneedles for methotrexate delivery to treat rheumatoid arthritis**

Qi Chen^a,#^, Chengyuan Wu^b,#^, Siwei Wang^b^, Qiang Wang^b^, Peiyun Wu^b^, Lei Wang^b,*^, Peiyu Yan^c,*^, Ying Xie^d,*^

*a. Faculty of Chinese Medicine ，Macau University of Science and Technology, Macao, China;*

*b.* *College of Pharmacy, Anhui University of Chinese Medicine, Hefei 230012, China;*

*c. Macau University of Science and Technology, Faculty of Chinese Medicine, State Key Laboratory of Quality Research in Chinese Medicines,Macao,China;*

*d.* *State Key Laboratory of Dampness Syndrome of Chinese Medicine, The Second Affiliated Hospital of Guangzhou University of Chinese Medicine, Guangzhou, China;*

Corresponding authors at: State Key Laboratory of Dampness Syndrome of Chinese Medicine, The Second Affiliated Hospital of Guangzhou University of Chinese Medicine, Guangzhou, China

E-mail addresses: leoxieying16@outlook.com(Ying Xie), pyyan@must.edu.mo(Peiyu Yan), wanglei@ahtcm.edu.cn(Lei Wang)

^#^These authors contributed equally to this study

E-mail addresses: caseychenqi2022@163.com(Qi Chen), 1219293070@qq.com(Chengyuan Wu).

**S1. Methods**

**S1.1 Glycyrrhizic acid grafting amount**

The absorbance of different concentrations of GA at 260 nm wavelength was measured, and the standard curve was established. The absorbance of GA-CDs and CDs at 260 nm was measured by ultraviolet spectrophotometer, and the grafting amount of GA in GA-CDs was calculated according to the standard curve.

**S2. Result**


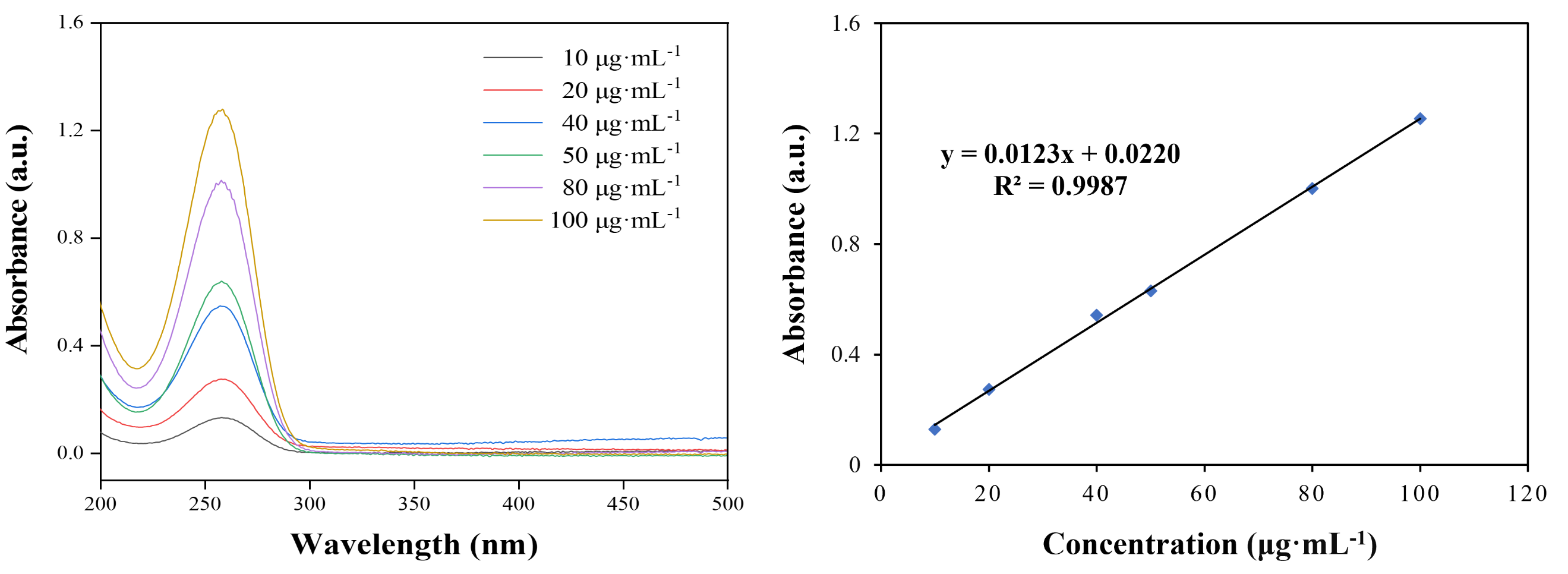


Fig. S1. UV spectra of different concentrations of GA


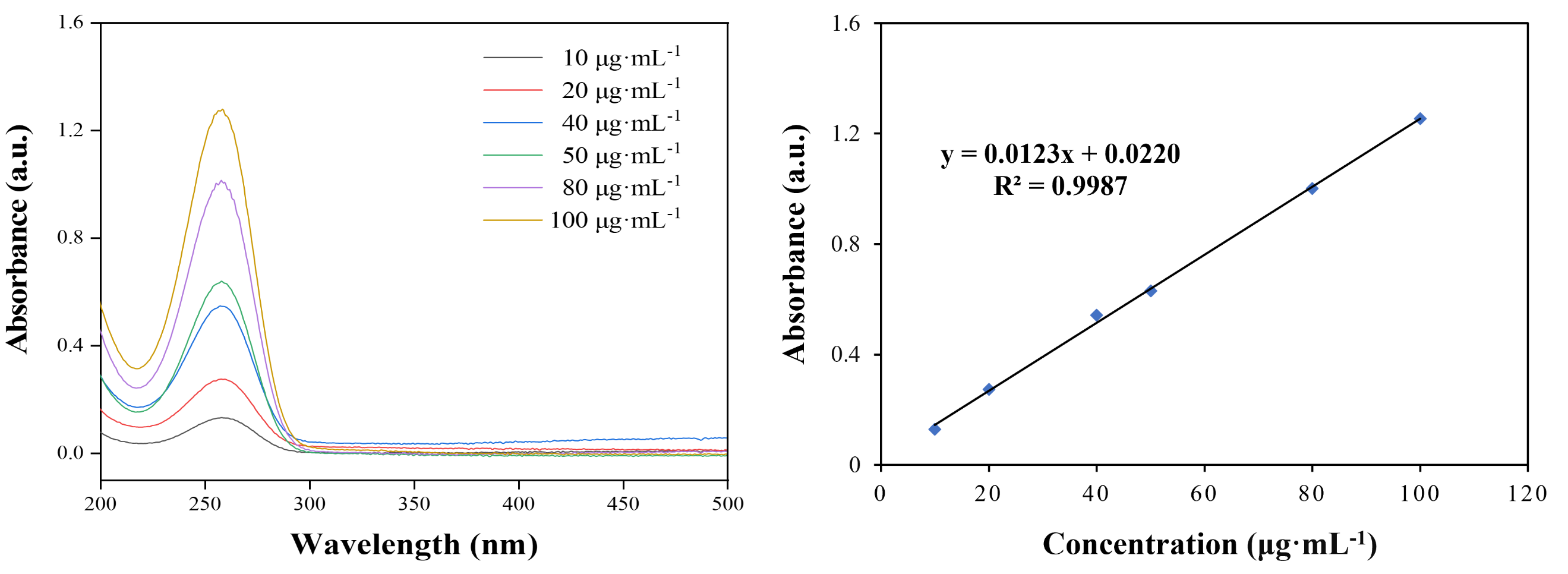


Fig. S2. Standard curve for determination of glycyrrhizic acid by ultraviolet spectrophotometry


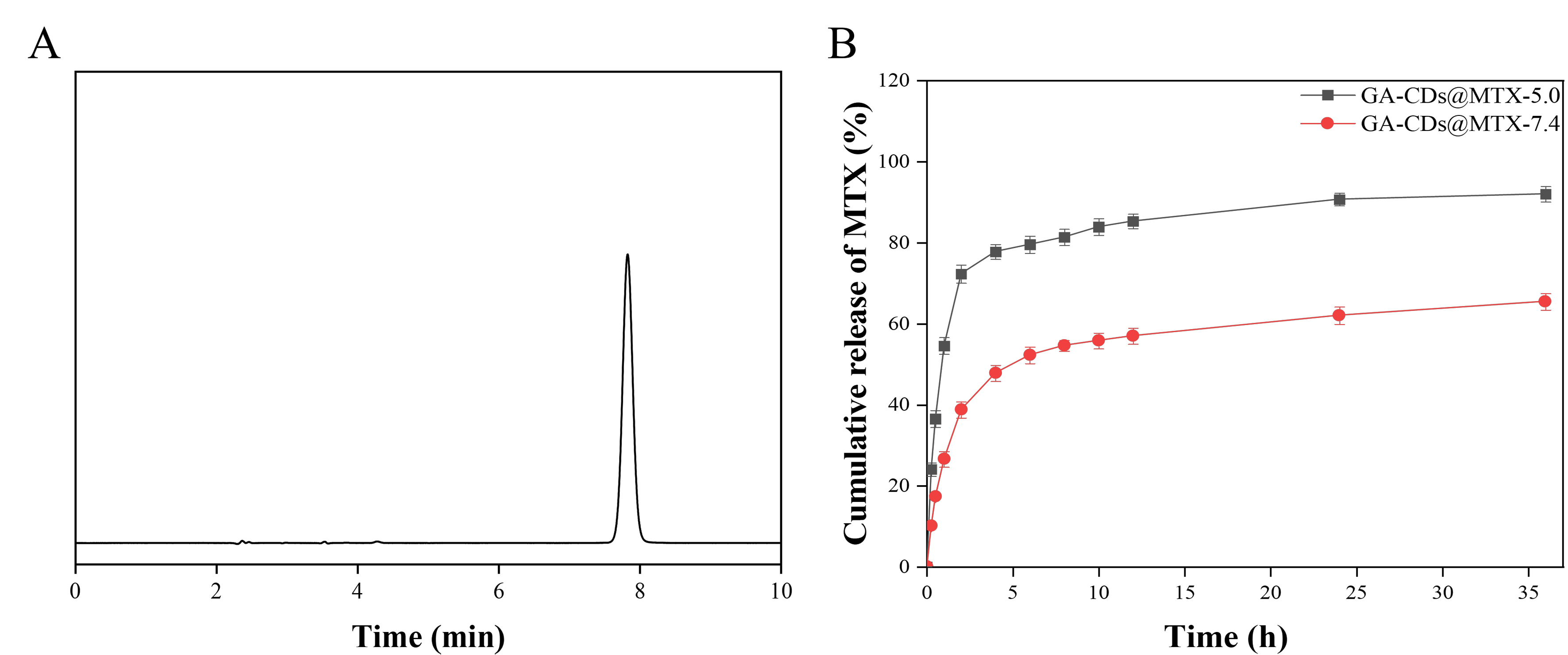


Fig. S3. High performance liquid chromatogram of MTX.


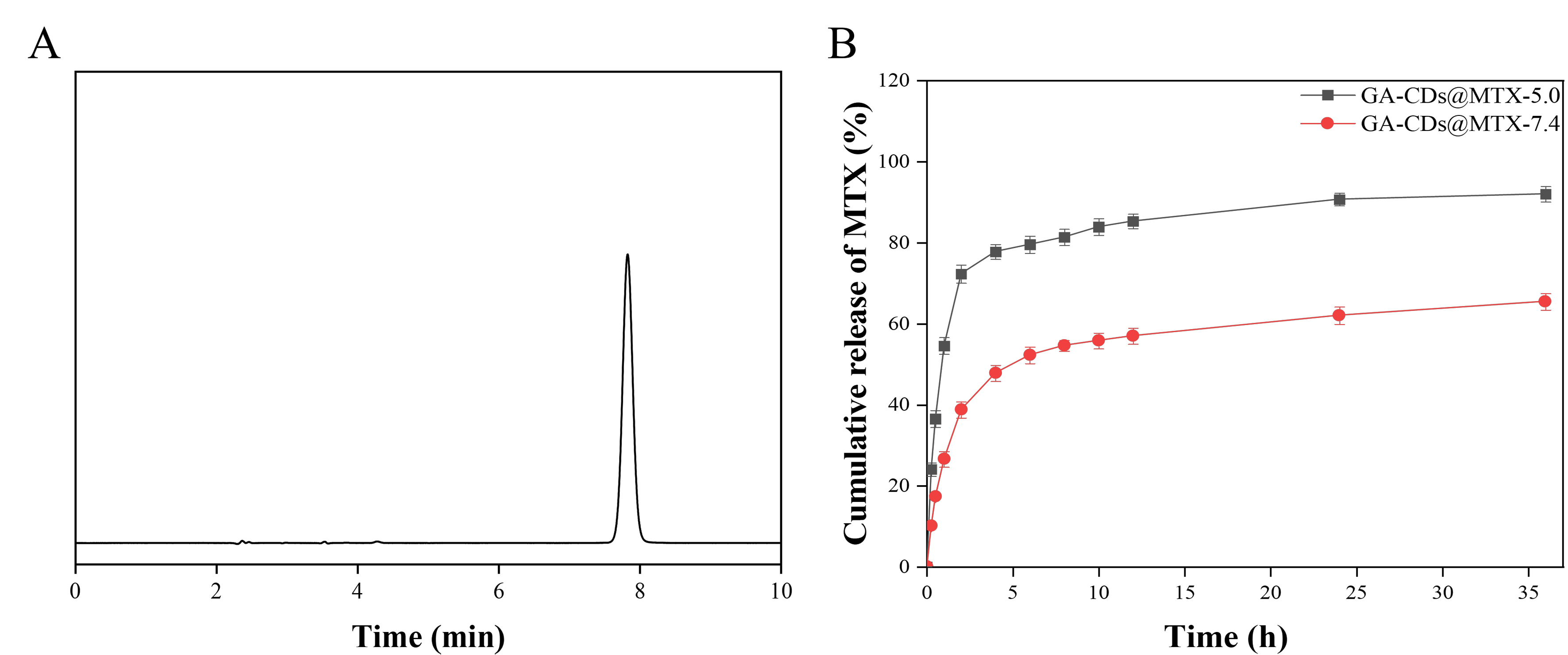


Fig. S4. In-vitro release profile of MTX from GA-CDs@MTX in PBS (pH 7.4 and pH 5.0) at 37 °C.
